# Catechins and Procyanidins of *Ginkgo biloba* Show Potent Activities towards the Inhibition of β-Amyloid Peptide Aggregation and Destabilization of Preformed Fibrils

**DOI:** 10.3390/molecules19045119

**Published:** 2014-04-22

**Authors:** Haiyan Xie, Jing-Rong Wang, Lee-Fong Yau, Yong Liu, Liang Liu, Quan-Bin Han, Zhongzhen Zhao, Zhi-Hong Jiang

**Affiliations:** 1 School of Chinese Medicine, Hong Kong Baptist University, Kowloon Tong, Kowloon, Hong Kong, China; 2 State Key Lab of Quality Research in Chinese Medicine, Macau Institute for Applied Research in Medicine and Health, Macau University of Science and Technology, Macau, China

**Keywords:** *Ginkgo biloba*, catechin, procyanidin, inhibition of aggregation, β-amyloid

## Abstract

Catechins and procyanidins, together with flavonoid glycosides and terpene trilactones, are three important categories of components in the standard extract of *Ginkgo biloba* leaves (EGb761). In this research, catechins and proanthocyanidins were found to exist in both the extract of Ginkgo leaves and Ginkgo products. By comparing with reference compounds, six of them were identified as (+)-catechin, (−)-epicatechin, (−)-gallocatechin, (−)-epigallocatechin and procyanidins B_1_ and B_3_. The activities of these polyphenols in the inhibition of Aβ42 aggregation and the destabilization of preformed fibrils were evaluated using biochemical assays, which showed that all six of the polyphenols, as well as a fraction of the extract of *Ginkgo biloba* leaves (EGb) containing catechins and procyanidins, exerted potent inhibitory activities towards Aβ42 aggregation and could also destabilize the performed fibrils. Catechins and procyanidins can therefore be regarded as the potent active constituents of EGb761 in terms of their inhibition of Aβ42 aggregation and destabilization of the fibrils. Although quantitative mass spectroscopic analysis revealed that the catechins and procyanidins are only present in low concentrations in EGb761, these components should be studied in greater detail because of their potent inhibitory effects towards Aβ42 aggregation and their ability to destabilize preformed fibrils, especially during the quality control of Ginkgo leaves and the manufacture of Ginkgo products.

## 1. Introduction

The standard extract of the leaves of *Ginkgo biloba* (EGb761) is current used clinically in Europe for the symptomatic treatment of impaired cerebral function in primary degenerative dementia syndromes such as Alzheimer’s disease (AD) dementia and vascular dementia. The abnormal production and aggregation of amyloid β peptide (Aβ) in the brain, as well as the deposition of fibrils are regarded as key steps in the onset of AD, and the development of therapeutic strategies aimed at preventing the aggregation of Aβ or promoting the destabilization of the preformed fibrils therefore represent viable approaches for the prevention and treatment of AD [1].

Our previous research in this area revealed that some of the flavonoid glycosides (FGs) isolated from EGb761 exhibited only moderate activity towards Aβ42 aggregation *in vitro*, whereas some other FGs and a range of terpene trilactones (TTLs) (*i.e.*, bilobalide and ginkgolides A, B and C) showed very little activity towards the same target [2]. Taken together, these results indicated that some other compounds may be responsible for the effect of the total extract. Catechins and proanthocyanidins have been reported to be important to the beneficial properties of EGb761, and compounds belonging to these structural classes make up 2% and 7% of the total extract content, respectively [3]. With this in mind, we became interested in identifying catechins and procyanidins of EGb761 and evaluating their activities towards the inhibition of Aβ42 aggregation and the destabilization of fibrils.

## 2. Results and Discussion

### 2.1. Identification of the Catechins and Procyanidins in EGb761

**Figure 1:**
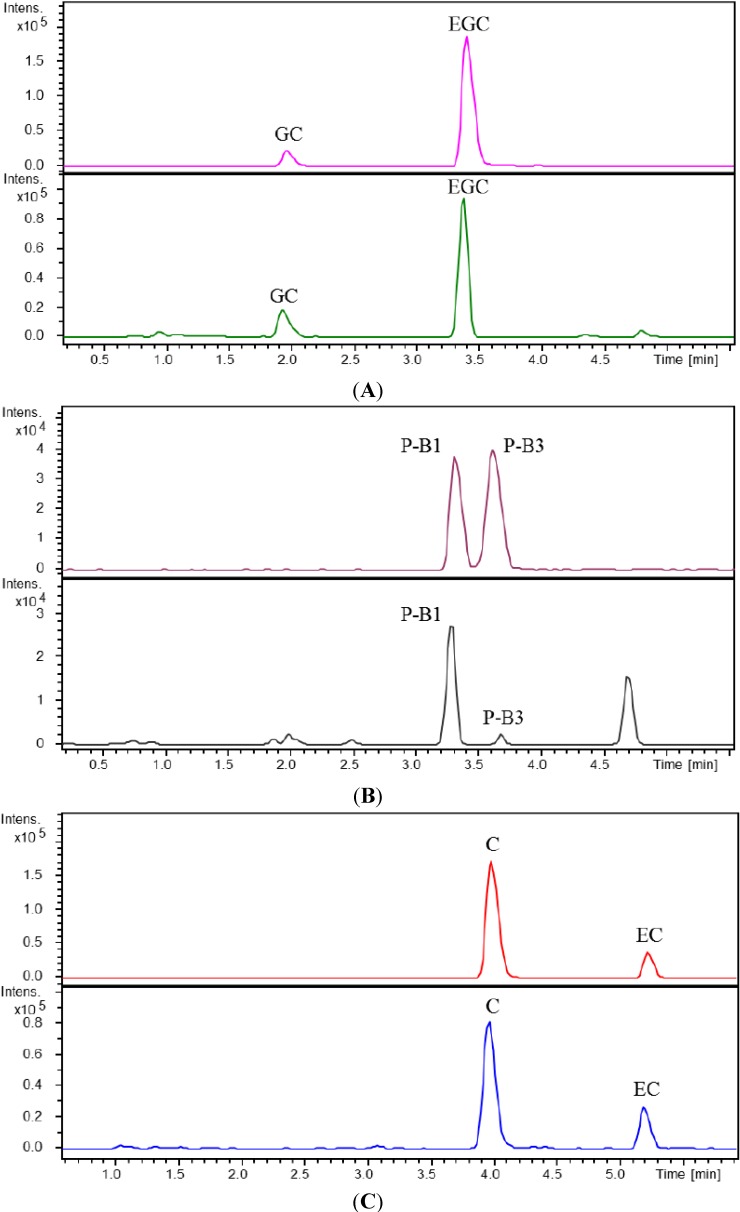
Extracted ion chromatograms (EIC) of GC and EGC (**A**); P-B_1_ and P-B_3_ (**B**); C and EC (**C**) in mixed standards (upper panel) and in tanakan^®^ tablets (lower panel).

**Figure 2:**
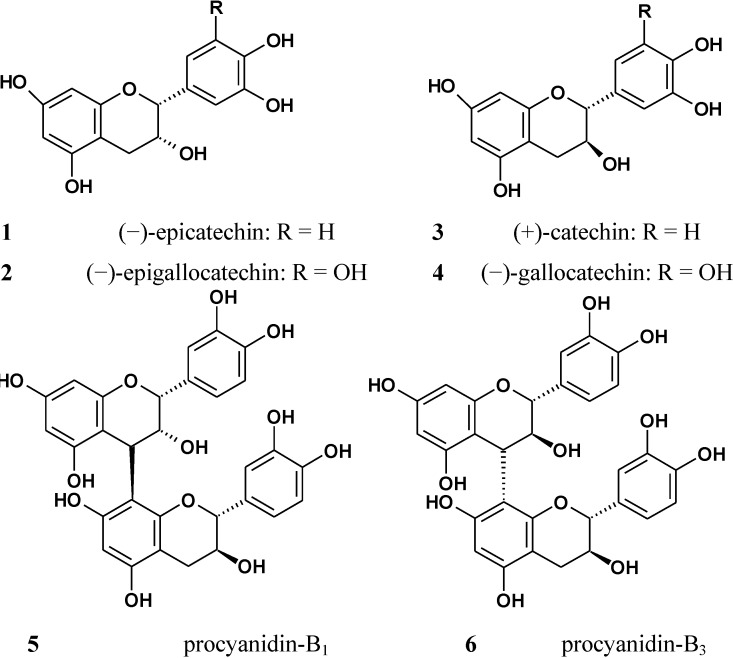
Structures of the catechins and procyanidins identified in EGb761.

Qualitative analyses were performed using ultra-performance liquid chromatography-electrospray ionization quadrupole time-of-flight mass spectrometry (UPLC-ESI-Q-TOF-MS), and revealed the presence of four catechins and four proanthocyanidins in tanakan^®^ tablets, a prescription medicine made from EGb761 and manufactured by European pharmaceutical companies. By comparing with reference compounds, the four catechins were identified as (+)-catechin (C), (−)-epicatechin (EC), (−)-gallocatechin (GC) and (−)-epigallocatechin (EGC). With regard to the four dimers, two of them were found to be consistent with the reference compounds and were consequently identified as procyanidins B_1_ (P-B_1_) and B_3_ (P-B_3_) (Figure 1). The two remaining compounds, however, could not be identified by such a comparison because of the lack of a reference compound, and they were provisionally identified as dimers of GC or EGC based on their accurate molecular weight. The structures of these six polyphenolic compounds are shown in Figure 2.

### 2.2. Bioactivity Assay of the Catechins and Procyanidins in Ginkgo

Using our previously reported method [2], we evaluated the inhibitory activities of these polyphenolic compounds (*i.e.*, C, EC, GC, EGC, P-B_1_ and P-B_3_) towards Aβ aggregation. All of these compounds exhibited significant inhibitory activity towards Aβ42 aggregation, with half maximal inhibitory concentration (IC_50_) values in the range of 3 to 18 μM (Table 1). Furthermore, the procyanidins were found to be more potent than the catechins in terms of their inhibition of Aβ42 aggregation. The activities of these compounds towards the destabilization of the performed fibrils were also evaluated using a previously reported method [4], and the results revealed that all of these compounds exhibited significant activity in this regard, with half maximal effective concentration (EC_50_) values in the range of 3 to 16 μM (Table 1). In general, procyanidins B_1_ and B_3_ showed much higher levels of activity than the catechins. Among all of the tested polyphenolic compounds, GC exerted the least influence in terms of its inhibition of Aβ aggregation, as well as the ability to destabilize fibrils.

**Table 1:** IC_50_ values for Aβ42 aggregation and EC_50_ values for destabilization of the preformed fibrils.

Compounds	IC_50_ on Aggregation (μM)	EC_50_ on Destabilization (μM)
C	14.93 ± 3.42	5.27 ± 1.26
EC	9.44 ± 0.24	5.99 ± 0.60
GC	17.54 ± 0.57	15.68 ± 3.19
EGC	8.51 ± 0.96	7.76 ± 0.94
P-B_1_	3.28 ± 0.46	3.51 ± 0.32
P-B_3_	3.54 ± 0.39	5.12 ± 0.75

These results were found to be in agreement with the review by Porat *et al.* [5], which reported that catechin and epicatechin could specially inhibit various amyloid proteins *in vitro*. Compared to the activities of another two categories of compounds existing in EGb 761, *i.e.*, FGs that possess moderate activities, with IC_50_ values ranging from 33 to 67 μM, and TTLs which only show very weak activities [2], catechins and procynidines exhibited much more potent inhibitory activity against Aβ aggregation. This strongly indicated the potentially important role of catechins and procyanidins in the inhibitory activity of EGb towards the Aβ aggregation.

### 2.3. Quantification of the Catechins and Procyanidins in the Extracts of Ginkgo

The potent inhibitory activities of these polyphenolic compounds towards Aβ aggregation and their ability to destabilize fibrils led us to investigate their contents in Ginkgo products. A review of the literature revealed that there were no methods available for the quantitative analysis of the catechins and procyanidins in EGb761, and it was therefore necessary to develop a new UPLC-Q-TOF-MS method to allow for the quantitative analysis of Ginkgo samples.

#### 2.3.1. Validation of UPLC-Q-TOF-MS Method

To begin with, we evaluated the stability of a standard solution containing the six compounds of interest. The results of this stability study revealed that the solution was only stable for approximately 2 h at room temperature. The addition of ascorbic acid to a solution of green tea catechins (pH 2.55) has been reported to enhance the stability for of these compounds for up to a month at room temperature [6]. With this in mind, the stability study was conducted a second time in the presence of ascorbic acid to determine its effect on the stability of the catechins and procyanidins. By comparing the stability of mixed standards solutions with different concentrations of ascorbic acid (*i.e.*, 0.1, 1.0 and 10.0 mM), it was found that the presence of 1 mM ascorbic acid or above rendered the solution stable for more than two days in the auto-sampler. Because the antioxidant effect of ascorbic acid led to a significant improvement in the stability of the compounds in solution, the mixed standard stock solutions were prepared in 20% methanol with 1 mM ascorbic acid.

The regression equations, linear ranges, lower limits of detection (LODs) and lower limits of quantification (LOQs) for the six analytes were determined using our newly developed method. As shown in Table 2, the data obtained using the new method indicated a good relationship between the concentrations of the analytes and their peak areas within the test ranges (*R^2^* > 0.9992). The LODs (S/*N =* 3) and LOQs (S/*N =* 10) were less than 0.005 and 0.017 µg/mL, respectively.

**Table 2:** Linearity and sensitivity properties of the analytes.

Compounds	Regression equation	*R^2^*	Linear range (µg/mL)	LOD (µg/mL)	LOQ (µg/mL)
C	Y = 641,968x + 21,451	0.9992	0.05–1.6	0.002	0.005
EC	Y = 685,413x + 5299.2	0.9994	0.02–1.0	0.005	0.017
GC	Y = 874,430x − 1298.7	0.9998	0.01–0.5	0.003	0.010
EGC	Y = 597,165x + 23,691	0.9994	0.05–1.6	0.003	0.010
P-B_1_	Y = 800,399x + 1111.8	0.9998	0.04–1.0	0.003	0.012
P-B_3_	Y = 2,000,000x − 10,522	0.9998	0.02–0.5	0.005	0.012

The validation process was evaluated by calculating the relative standard deviations (RSDs), and the results confirmed that this method showed good precision and accuracy. As shown in Table 3, the overall intra-day and inter-day variations (RSD) of all the six analytes were less than 3.74% and 4.03% (*n =* 5), respectively. The repeatability of the method was evaluated by analyzing a sample taken from one batch of EGb six times in quick succession, and the RSDs were found to be less than 3.80%. Recovery studies were also performed by spiking a mixed standard solution (the same concentration as the sample) into the powder of the EGb sample six times in quick succession. The recoveries were found to be in the range of 95.76%–103.56%, and all of the RSDs were less than 1.53% for each analyte.

**Table 3:** Precision, repeatability and recovery properties of the analytes.

Compounds	Precision (RSD,%, *n =* 5)		Repeatability ( *n =* 6)		Recovery (%, *n =* 6)		
	Intra-day	Inter-day	Concentration (ng/mg)	RSD (%)	Range	Mean	RSD
C	2.31	3.16	12.54	3.80	97.53–100.97	99.53	1.53
EC	1.07	2.89	3.03	1.66	97.83–99.88	98.96	0.80
GC	3.74	3.69	1.58	1.18	98.58–101.24	99.67	0.88
EGC	2.57	2.72	8.06	1.76	100.74–103.56	101.92	1.28
P-B_1_	1.92	4.03	2.74	2.37	97.98–100.38	99.33	1.07
P-B_3_	2.69	3.79	1.69	2.85	95.76–98.43	97.26	1.03

Based on these results, it was clear that our newly developed UPLC-Q-TOF-MS method was an accurate and sensitive enough method for the quantitative analysis of the six polyphenolic compounds in Gingko extracts.

#### 2.3.2. Quantitative Analysis of the Catechins and Procyanidins in Gingko Extracts

**Figure 3:**
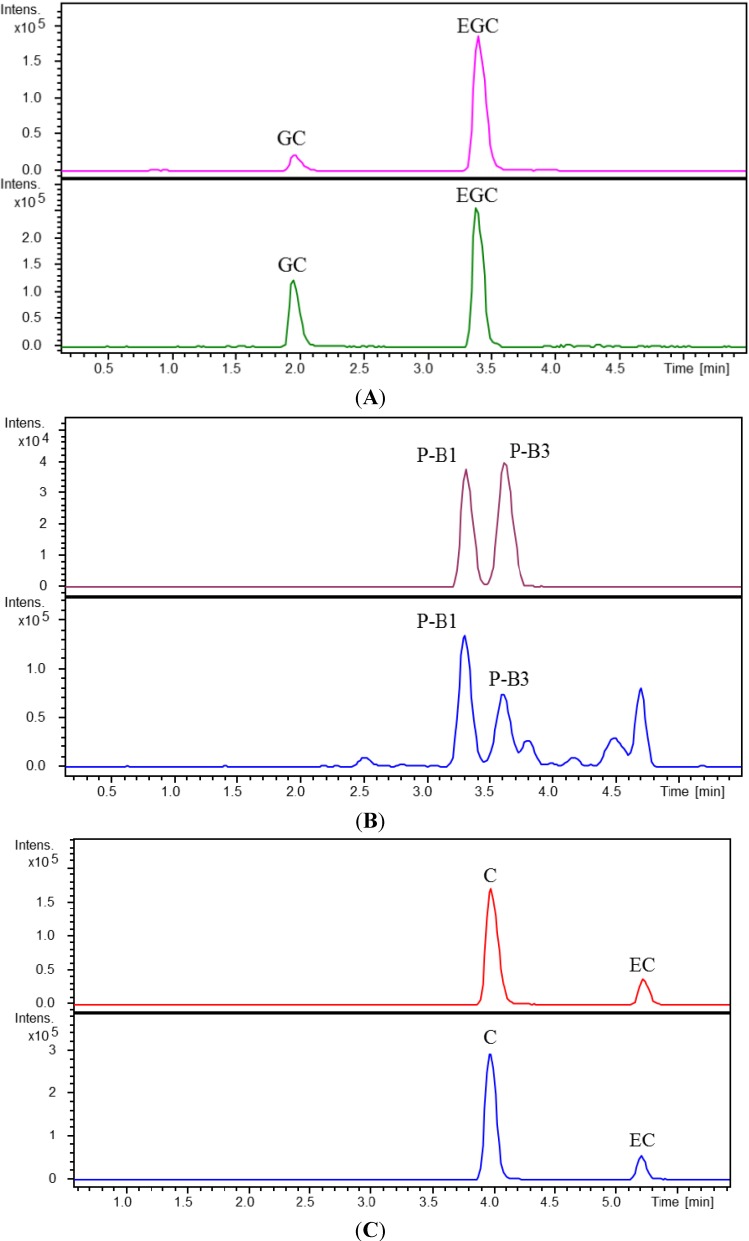
Extracted ion chromatograms (EIC) of GC and EGC (**A**); P-B_1_ and P-B_3_ (**B**); C and EC (**C**) in mixed standards (upper panel) and in an extract of the Ginkgo leaves (lower panel).

Seven samples of extracts from Ginkgo leaves and products (Table 4) were quantitatively analyzed for four catechins and two procyanidins using our newly developed UPLC-Q-TOF-MS method (Table 5). The extracted ion chromatograms (EIC) of the six compounds under investigation in the mixed standards and the Ginkgo extract were shown in Figure 3.

**Table 4:** Samples for quantitative analysis.

Sample	Description
S1	Extract, extracted from tanakan^®^ tablets (manufactured by Beaufour Ipsen Industrie, Dreux, France and purchased from Watsons, HK)
S2	Extract, extracted from tanakan^®^ tablets (manufactured by Beaufour Ipsen Industrie, Dreux, France and purchased from Tianjin, China)
S3	Extract, extracted from Ginaton^®^ tablets (manufactured by Dr. Willmar Schwabe GmbH & Co. KG, Karlsruhe, Germany and purchased from Tianjin, China)
S4	Extract, extracted from Ginaton^®^ tablets (manufactured by Dr. Willmar Schwabe GmbH & Co. KG, Karlsruhe, Germany and purchased from Tianjin, China)
S5	Extract of Ginkgo leaves collected from Shandong Province, China
S6	Extract of Ginkgo leaves collected from Hunan Province, China
S7	Extract purchased from Ningbo Traditional Chinese Pharmaceutical Co., Ltd., Zhejiang Province, China

**Table 5:** Catechin and procyanidin contents of the extracts (*n =* 2).

Sample	Contents (ng/mg)						
	C	EC	GC	EGC	P-B_1_	P-B_3_	Sum
S1	17.08	4.30	2.56	19.25	5.51	1.55	50.26
S2	13.04	3.09	2.15	8.20	2.81	1.73	31.02
S3	4.00	0.95	0.59	1.81	1.50	1.09	9.93
S4	14.12	3.33	2.03	13.38	4.83	1.88	39.55
S5	0.59	nd	nd	0.13	1.30	0.91	2.93
S6	8.59	2.21	0.79	10.19	4.28	1.83	27.89
S7 ^a^	3.04	1.66	0.60	1.30	0.36	0.10	7.06

^a^ contents (µg/mg); nd: not detected.

The results revealed that contents of these samples varied considerably. Both of the commercially available ginkgo tablets, tanakan^®^ and Ginaton^®^, showed large batch-to-batch variations in their catechin and procyanidin contents, despite good batch-to-batch repeatability in terms of their FG and TTL contents which varied in the range of 9.20%–11.10% and 7.46%–8.55%, respectively [2]. Furthermore, there was almost a 10-fold difference between the contents of two leaves that were grown in different areas.

### 2.4. Preparation, Quantitative Analysis and Bioactivity Assay of Fractions containing Catechins and Procyanidins from EGb

Because we have demonstrated that these potent polyphenolic compounds (*i.e.*, C, EC, GC, EGC, P-B_1_ and P-B_3_) are present in only minor concentrations in the Ginkgo extracts and Ginkgo products (Table 5), it was envisaged that a fraction enriched with catechins and procyanidins would be useful for assessing the inhibitory activities of these compounds towards Aβ42 aggregation. With this in mind, the total extract of Ginkgo leaves was separated into several fractions and sub-fractions, which were enriched with catechins and procyanidins. The total extract and fractions were then subjected to quantitative analysis and biochemical evaluation, and samples containing relatively high levels of catechins and procyanidins were evaluated in terms of the inhibitory effect on Aβ42 aggregation.

#### 2.4.1. Preparation of Fractions containing Catechins and Procyanidins from EGb

A total extract sample (S7) was subjected to MCI-gel column chromatography and gave four fractions (*Fr. A–D*) in yields of 1.2%, 7.2%, 62.6% and 10.7%, respectively. Catechins and procyanidins were found to be abundant in *Fr. B* (15%–40% MeOH eluent), whereas FGs were mainly found in *Fr. C* (40%–70% MeOH eluent). TTLs were found in both *Fr. B* and *Fr. C*. Chemical composition of *Fr. D* was quite similar to that of *Fr. C* with the only difference in relative levels of individual constituents.

*Fr. B* was then subjected to sequential purification over ODS and TSK columns to yield a fraction enriched in catechins and procyanidins (*Fr. Ps*) with an overall yield of 0.97%. *Fr. C* and a sub-fraction of *Fr. B* were purified by column chromatography over successive ODS, TSK and Sephadex LH-20 columns to give fractions enriched in TTLs and FGs (*Fr. Ts* and *Fr. Fs*, respectively). Recovery yields of FGs, TTLs, as well as catechins and procyanidins in each fraction derived from EGb were calculated on the basis of quantitative analysis of corresponding components (Table 6).

**Table 6:** Recovery yield of each fraction from EGb.

Recovery yield (%)			
	FGs	TTLs	catechins and procyanidins
*Fr. B*	/	/	65.16
*Fr. C*	84.48	78.62	/
*Fr. Fs*	82.37	/	/
*Fr. Ts*	/	46.18	/
*Fr. Ps*	/	/	63.66

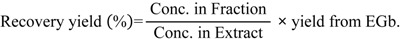

#### 2.4.2. Quantitative Analysis of the Fractions and the Extract

**Figure 4:**
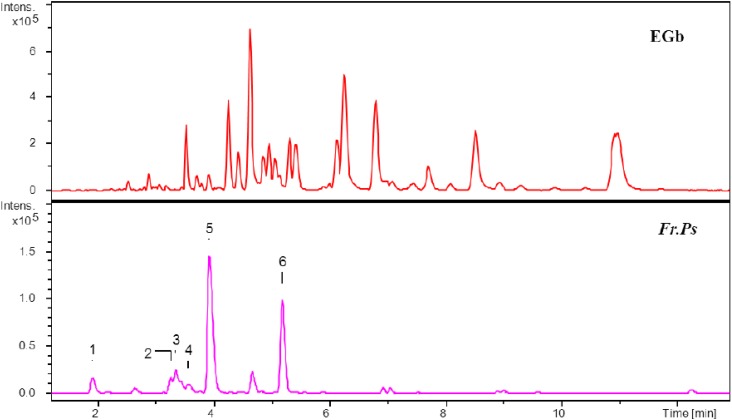
Total ion chromatograms (TIC) of the total extract of ginkgo leaves and the fraction enriched in catechins and procyanidins (*Fr. Ps*). (1: GC, 2: P-B_1_, 3: EGC, 4: P-B_3_, 5: C, 6: EC).

The specific fraction (*Fr. Ps*) contained six polyphenols, namely C, EC, GC, EGC, P-B_1_ and P-B_3_ (Figure 4), whereas other fractions, except for *Fr. B*, were not found to contain these constituents. Therefore we proceeded to determine the catechin and procyanidin contents of the two fractions (*Fr. B* and *Fr. Ps*) by using our newly developed analytical method. The results were shown in Table 7.

**Table 7:** Analytical results for the catechins and procyanidins contents in the fractions and the extract.

Sample	Contents (µg/mg)						
	C	EC	GC	EGC	P-B_1_	P-B_3_	Sum
Extract	3.04	1.66	0.60	1.30	0.36	0.10	7.06
*Fr. B*	30.31	16.62	3.99	8.17	3.69	1.10	63.89
*Fr. Ps*	235.34	134.93	22.10	42.78	21.00	7.21	463.36

The results revealed that the catechin and procyanidin content of the extract increased from 0.7% to 6.4% in *Fr. B* following the first fractionation. Further fractionation led to further increases in the content, which reached 46.3% in *Fr. Ps*. This increase represented a 66-fold enrichment over the content observed in the initial extract, and indicated that *Fr. Ps* contained a high concentrated of catechins and procyanidins. In addition, using our previously reported method [2], contents of FGs in the extract, *Fr. C* and its sub-fraction *Fr. Fs*, were determined to be 16.7%, 22.5% and 48.2%, respectively. While TTLs represented 14.2%, 17.8% and 72.3% of the overall weight of the extract, *Fr. C* and its sub-fraction *Fr. Ts*, respectively.

#### 2.4.3. Bioactivity Assay of the Fractions and the Extract

With an enriched sample of the catechins and procyanidins in hand, we proceeded to evaluate its anti-aggregation effect towards Aβ42 aggregation (Table 8). The effect of other fractions and the extract was evaluated simultaneously for comparison.

**Table 8:** IC_50_ values of different fractions from EGb on Aβ42 aggregation.

Sample		Mean ± SD (µg/mL)
Extract		14.24 ± 1.00
Preliminary fraction	*Fr. A*	13.62 ± 0.15 ^††^
	*Fr. B*	4.98 ± 0.05 ^**^
	*Fr. C*	15.15 ± 0.18 ^††^
	*Fr. D*	18.75 ±1.64 ^*^^,^^††^
Sub-fraction	*Fr. Ps*	2.22 ± 0.11 ^**^
	*Fr. Ts*	136.2 ± 5.49 ^**^^,^^##^
	*Fr. Fs*	37.26 ± 2.03 ^**^^,^^##^

^*^ *p* < 0.05, ^**^ *p* < 0.01, *vs.* Extract; ^††^ *p* < 0.01, *vs.* *Fr. B*; ^##^ *p* < 0.01, *vs.* *Fr. Ps*.

As shown in Table 8, all of the fractions derived from the extract (S7), except for *Fr. Ts*, exhibited some inhibitory activity towards Aβ42 aggregation, with IC_50_ values ranging from 2.22–37.26 µg/mL. When we considered these data in greater detail, it was clear that *Fr. B* showed the most potent activity of the four preliminary fractions, which implied that the most effective constituents were present in *Fr. B*. *Fr. Ps*, the sub-fraction derived from *Fr. B* and enriched with catechins and procyanidins showed much greater inhibitory activity than the other two sub-fractions which contained flavonoids (*Fr. Fs*) and terpene trilactones (*Fr. Ts*) (^##^ *p* *<* 0.01). Taken together, *Fr. B* and its subfraction, *Fr. Ps*, both contained catechins and procyanidins, exhibited the greatest inhibitory activities. The results provided further evidence to suggest that catechins and procyanidins play a critical role in the anti-aggregation effect of EGb761. In addition, *Fr. Fs* showed moderate effect and *Fr. Ts* barely exerted effect, in accordance with the effect of individual compounds of flavonoid glycosides and terpene trilactones [2].

## 3. Experimental

### 3.1. Reagents

Catechin hydrate (C, purity ≥ 99%) and gallocatechin (GC, purity ≥ 98%) were purchased from Nagara Science Co., Ltd. (Gifu, Japan). Epicatechin (EC) and epigallocatechin (EGC) (purity > 98%) were purchased from Winherb Medical Science (Shanghai, China) Procyanidin B_1_ (P-B_1_) and procyanidin B_3_ (P-B_3_) were isolated and structurally elucidated in our lab before (purity > 95%). The Aβ42 peptide was produced by Bachem AG (Bubendrof, Switzerland) with purity greater than 95%, as determined by HPLC. Thioflavin T (ThT), 1,1,1,3,3,3-hexafluoro-2-propanol (HFIP) and dimethyl sulfoxide (DMSO) were obtained from Sigma-Aldrich (St. Louis, MO, USA). Trifluoroacetic acid (TFA) was purchased from International Laboratory (San Francisco, CA, USA).

### 3.2. Polymerization Assay and Destabilization Assay

The polymerization assay was performed as previously described in the literature [2]. For single compounds, the reaction mixture contained 25 μM Aβ42, 0.3, 1.0, 3.0, 10, or 30 μM C, EC, GC, EGC, P-B_1_, or P-B_3_, 1% DMSO, 50 mM phosphate buffer (pH 7.5) and 100 mM NaCl. Prior to being tested, the compounds were dissolved in DMSO at concentrations of 30, 100 and 300 μM, and 1.0 and 3.0 mM, and then added to the reaction mixture to final concentrations 0.3, 1.0, 3.0, 10, and 30 μM, respectively. For the extract and its fractions, different concentration sequences were incubated in the reaction mixture. With the exception of one sequence (*i.e.*, 3.0, 10, 30, 100 and 200 μg/mL) for *Fr. Ts* and another (*i.e.*, 0.3, 1.0, 3.0, 10, 30 μg/mL) for *Fr. Ps*, one concentration sequence (*i.e.*, 1.0, 3.0, 10, 30 and 100 μg/mL) was used for the extract and all of the other fractions.

The destabilization assay used to evaluate the activities of the polyphenolic compounds towards the destabilization of preformed fibrils was performed as described by Ono *et al.* [4]. Briefly, a reaction mixture containing 25 μM Aβ42 and 50 mM phosphate buffer (pH 7.5 and 100 mM NaCl) was incubated on a PCR system for 24 h at 37 °C to form fibrils. All of the test compounds (*i.e.*, C, EC, GC, EGC, P-B_1_, and P-B_3_) were initially dissolved in DMSO at concentrations of 30, 100 and 300 μM, and 1 and 3 mM, before being added to the reaction mixture to give final concentrations of 0.3, 1.0, 3.0, 10, and 30 μM, respectively. The resulting mixtures were continued the incubation of 24 h at 37 °C, and then subjected to fluorescent measurements as in the polymerization assay.

The fluorescence measurements were recorded on an Envision^®^ 2104 Multilabel Reader (Perkin Elmer, Waltham, MA, USA), with optimized excitation and emission wavelengths of 430 (BW 24 nm) and 470 nm (BW 24 nm), respectively. The reaction mixture contained 5 μM ThT and 50 mM of glycine-NaOH buffer (pH 8.5). The IC_50_ and EC_50_ values were calculated using the GraphPad Prism 5 software. All results were expressed as the mean ± SEM, and statistical analyses were performed using the Student’s *t* test.

### 3.3. Quantitative Analysis of the Catechins and Procyanidins in Ginkgo Extracts by UPLC-Q-TOF-MS

Mixed stock solutions containing C (50 µg/mL), EC (10 µg/mL), GC (5 µg/mL), EGC (50 µg/mL), P-B_1_ (10 µg/mL) and P-B_3_ (5 µg/mL) were prepared in 20% methanol (with 1 mM ascorbic acid). Working standard solutions for the calibration curves were prepared by diluting the stock solution with 20% methanol to the following concentrations: 0.01–0.5 µg/mL for GC, P-B_3_; 0.02–1.0 µg/mL for P-B_1_; 0.05–1.6 µg/mL for C and EGC; and 0.01–1.0 µg/mL for EC. The standard solutions were stored at −20 °C prior to be analyzed.

A portion (50 mg) of the Ginkgo extract was accurately weighed into a 2 mL tube followed by 1 mL of 20% methanol, and the resulting mixture was briefly agitated to allow for mixing. The mixture was then sonicated for 10 min and filtered through a 0.22 µm PTFE filter to yield a sample solution for analysis.

UPLC was performed on an Acquity UPLC system (Waters Corp, Milford, MA, USA) equipped with a binary solvent delivery system and a sample manager. The UPLC system was coupled to a Bruker MicroTOF mass spectrometer with an ESI source (Bruker Daltonics, Billerica, MA, USA). Data acquisition and analysis were performed using the Hystar software (Bruker).

UPLC analysis was performed on an Acquity UPLC HSS C18 column (2.1 × 150 mm, 1.8 μm) with a mobile phase consisting of 0.1% (*v/v*) formic acid in water (A) and 0.1% (*v/v*) formic acid in acetonitrile (B). The chromatography was performed according to the following gradient elution procedure: 0–2 min, 6.0%–7.8% B; 2–15 min, 7.8%–40% B; 15.1–18 min, 100% B; 18.1–20 min, 6% B. The flow rate for the UPLC analysis was set at 0.35 mL/min, with a column temperature of 40 °C. An injection volume of 5 µL was used for quantitative analysis.

The ESI-MS data were acquired in the negative ion mode using the following MS parameters: end plate offset, −500 V; capillary voltage, 4500 V; collision energy, 6.0 eV; nebulizing gas (N_2_) pressure, 2.0 bar; drying gas (N_2_) flow rate, 8.0 L/min; drying gas temperature, 180 °C; and Mass range, *m/z* = 100–1600.

The presence of C, EC, EGC, GC, P-B_1_ or P-B_3_ in the sample was identified by comparing its retention time and the mass spectrum with those of the standards. The extracted ion chromatograms (EICs) at the *m/z* 305.06 for the [M–H]^−^ ions of GC and EGC, *m/z* 577.13 for the [M–H]^−^ ions of P-B_1_ and P-B_3_, and *m/z* 289.07 for the [M–H]^−^ ions of C and EC were integrated and the peak areas were used for quantification.

### 3.4. Preparation of Fractions containing Catechins and Procyanidins from EGb761

The extract of *Ginkgo biloba* leaves (S7, 3.0 g) was purified by column chromatography over MCI-gel CHP 20P (75–100 mm, Mitsubishi Chemical Co., Ltd., Tokyo, Japan). The column was successively eluted with water containing 5%, 10%, 15%, 20%, 25%, 30%, 40%, 50%, 60%, 70%, 80%, 90% and 100% MeOH, which resulted in four fractions (*Fr. A–D*) being collected. Catechins and procyanidins were found in *Fr. B* (15%–40% MeOH eluent) and the FGs were mainly found in *Fr. C* (40%–70% MeOH eluent). The TTLs were found in both *Fr. B* and *Fr. C*.

*Fr. B* (200 mg) was purified over a ODS (Davisil, 35–60 μm, Grace, Columbia, MD, USA) column eluting with an increasing gradient of methanol in water (from 0%–80% MeOH). Catechins and procyanidins were found in *Fr. B2* (from 20%–35% MeOH). *Fraction B2* (151 mg) was further purified over a Toyopearl HW-40F (Tosoh Corporation, Japan) column eluting with an increasing gradient of methanol in water (from 0%–60% MeOH). Fraction *Fr. B2-2* was eluted with a 40%–60% mixture of MeOH to give a fraction abundant in catechins and procyanidins (*Fr. Ps*, 27 mg).

**Figure 5:**
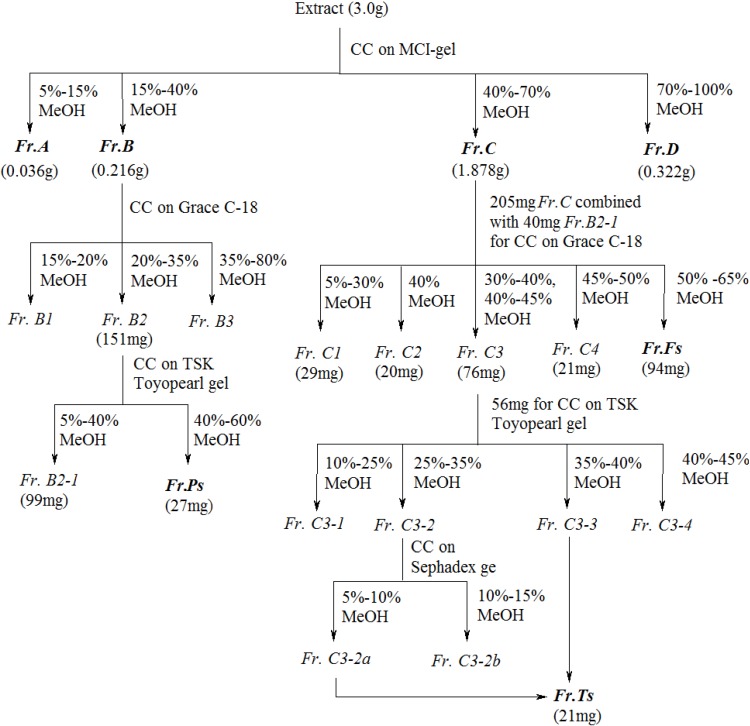
Flowchart showing the fractionation of catechins and procyanidins, FGs and TTLs from EGb. (CC: column chromatography; *Fr.*: fraction).

*Fr. C* (205 mg) and *Fr. B2-1* (40 mg, a portion of the material eluted with 5%–40% MeOH) were combined and purified over a Grace C-18 column eluting with an increasing gradient of methanol in water (from 0%–80% MeOH). *Fr. C5* (from the 50%–65% MeOH eluent) was enriched in FGs (*Fr. Fs*, 94 mg), whereas *Fr. C3* (*i.e.*, the combination of fractions from the 30%–40% and 40%–50% MeOH eluents) was enriched in TTLs. *Fr. C3* (76 mg) was purified over a Toyopearl HW-40F column eluting with an increasing gradient of methanol in water (*i.e.*, from 5%–50% MeOH) to remove some flavonoid compounds. *Fr. C3-3* (eluted with 35%–45% MeOH) mainly consisted ginkgolides A and B, and bilobalide, whereas *Fr. C3-2* (eluted with 25%–35% MeOH) contained of bilobalide, ginkgolide C and some flavonoids. This fraction was then purified over a Sephadex LH-20 (Aldrich Chemical Co., Inc., Milwaukee, WI, USA) column eluting with methanol and water to give pure bilobalide and ginkgolide C. *Fr. C3-2a* and *Fr. C3-3* were combined to give the TTLs (*Fr. Ts*, 21 mg) (Figure 5).

Thin layer chromatography (TLC) and UPLC-Q-TOF-MS analysis were used for the detection of the targeted compounds. TLC was conducted on silica gel 60 F254 HPTLC aluminium sheets (Merck, Darmstadt, Germany) while UPLC-Q-TOF-MS analyses were performed on the same equipment as that used above for the quantitative analyses, as well as being conducted under the same parameters, except for the gradient elution procedure. The gradient elution procedure for the qualitative analysis was as follows: 0–2 min, 6.0%–7.8% B; 2.0–12 min, 7.8%–18% B; 12–13 min, 18%–100% B; 18–20 min, 6.0% B. The polyphenolic compounds in the samples were identified by comparing their retention times and mass spectra with those of the standards. EICs at *m/z* 305.06 for the [M–H]^−^ ions of GC and EGC, *m/z* 577.13 for the [M–H]^−^ ions of P-B_1_ and P-B_3_, and *m/z* 289.07 for the [M–H]^−^ ions of C and EC were integrated. EICs at *m/z* 609.12 for the [M–H]^−^ ion of the dimer of GC or EGC (*i.e.*, GC-GC, EGC-EGC or GC-EGC), and *m/z* 593.13 for the [M–H]^−^ ion of the dimers of C(EC)-GC(EGC) were selected for the identification of possible procyanidins in EGb761.

## 4. Conclusions

Six polyphenolic compounds in the extract of *Ginkgo biloba* leaves were identified as (+)-catechin, (−)-epicatechin, (−)-gallocatechin, (−)-epigallocatechin, and procyanidins B_1_ and B_3_ through a comparison of their analytical data with those of the authentic reference compounds. These compounds were found to exhibit potent inhibitory activities towards Aβ42 aggregation, as well having a destabilizing effect on preformed fibrils. The procyanidins exhibited stronger effects than catechins in terms of their inhibition of Aβ42 aggregation and ability to destabilize preformed fibrils.

Furthermore, in an attempt to investigate the roles of catechins and procyanidins in the anti- aggregation effect of the Ginkgo extract, we isolated fractions enriched in these compounds from EGb and then subjected to a biochemical assays *in vitro*. The results revealed that a fraction abundant in catechins and procyanidins (*i.e.*, *Fr. Ps*) exerted a much greater level of inhibitory activity towards Aβ42 aggregation than the original extract and the other fractions enriched with FGs and TTLs (*i.e.*, *Fr. Fs*, *Fr. Ts*). These results indicated that catechins and procyanidins play an important role in the inhibitory activity of EGb towards Aβ42 aggregation. This research has therefore for the first time provided some insight into the potential application of these polyphenolic compounds towards the inhibition of Aβ42 aggregation and the destabilization of preformed fibrils. Substantial studies have revealed potential neuroprotective activities of catechins in tea [7,8,9,10,11]. Phase Ⅱ clinical trial of epigallocatechin-3-gallate (EGCG) on neurodegenerative disorders is being conducted [12]. Therefore, it is reasonable to indicate that these polyphenols existed in EGb761 are another category of constituents contributing to the neuroprotective effect of EGb761.

Although catechins and procyanidins are present at only minor concentrations in EGb and its products, based on the results of our UPLC-Q-TOF-MS quantitative analyses, further studies should be conducted to develop a deeper understanding of the potent effects of these compounds towards the inhibition of Aβ42 aggregation and the destabilization of preformed fibrils during the quality control process involved in the manufacture of Ginkgo leaves and products.
